# Reveal the differences in flavor of five varieties of black teas via sensory evaluation, UPLC, and GC × GC-QTOFMS approaches

**DOI:** 10.1016/j.fochx.2025.103083

**Published:** 2025-10-03

**Authors:** Mengcong Zhang, Xiaoshuang Lu, Peidi Zhang, Guangcai Li, Dongfang Li, Han Xie, Jian'’an Huang, Sheng Zhang, Zhonghua Liu, Ailing Liu

**Affiliations:** a Key Laboratory of Tea Science of Ministry of Education, Hunan Agricultural University, Changsha, Hunan 410128, China; b National Research Center of Engineering and Technology for Utilization of Botanical Functional Ingredients, Changsha, Hunan 410128, China; c Yuelushan Laboratory, Changsha 410128, China; d National Key Laboratory for Tea Plant Germplasm Innovation and Resource Utilization, Changsha 410128, China; e College of Bioscience and Biotechnology, Hunan Agricultural University, Changsha 410128, China.

**Keywords:** Black tea, Medium commercial quality, Sensory characteristics, Key flavor compounds, Aroma wheel

## Abstract

Commercial black teas vary widely, yet the formation mechanisms of their distinct flavors remain unclear. Here, five different varieties of black teas with medium commercial quality were collected and characterized by integrating flavoromics and chemometric approaches. Soluble sugar, gallocatechin gallate, free amino acids, caffeine, and flavonoids were identified as characteristic taste components. Among 165 identified volatiles, with 51 compounds as key odorants contributing to the diverse aromas of black teas. The 21, 22, 37, 25 and 22 aroma-active compounds were smelled in ZY, QM, ZS, FQ, and HP, respectively. The VIP, ROAV and GC-O results indicated that linalool, phenylethyl alcohol, linalool oxide I and (*E*)-furan linalool oxide were important aroma-active compounds present in all five black teas. Heptanal characterized FQ, while *α*-cubebene and *trans*-calamenene characterized HP and ZS. The results provide a basis for the study of flavor property and quality control of black tea.

## Highlights

• Sensory evaluation reveals significant differences in the flavor.
• Key flavor compounds in five different varieties of black teas were identified.
• The aroma wheel of five different varieties of black teas was established.

## Graphical abstract

**Figure:**
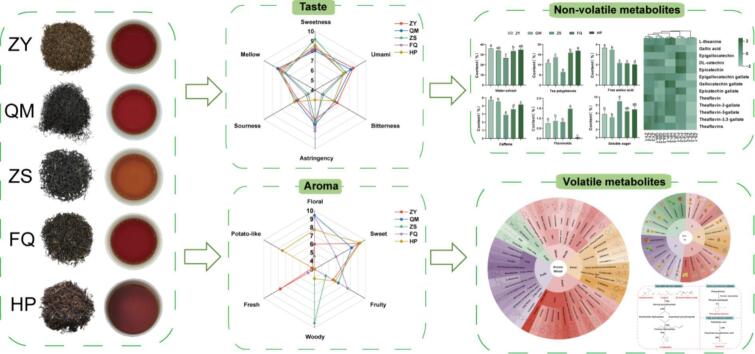

## 1 Introduction

Black tea is one of the world's most popular beverages, constitutes approximately 75 % of total tea consumption and represents the predominant category in both production and consumption among the six major tea types (Ouyang et al., 2024). Based on the appearance, color, aroma, taste and leaf bottom of tea products, black tea can be classified into various grades (Peng et al., 2023). Generally, the flavor profiles of black teas differ significantly across various grades. In the market, the price of black tea varies on the basis of its quality grade, of which can vary by several times, or even dozens of times (Zhu et al., 2019). There is quality fraud in the tea market, and the quality of tea often does not correspond to its price. Consistent product quality will lead to repeat purchases, which in turn increases consumption (Su et al., 2022). A systematically and comprehensively investigation to the quality of tea in different price ranges is of great significance for standardizing the pricing of the tea market and protecting the rights and interests of consumers. The recent report on China's tea production and sales revealed that black tea priced between $45 and $55 per kilogram accounts for the largest share of consumption in China. Here we define black teas in this price range as medium commercial quality and exhibiting distinct flavor characteristics. Zunyi black tea have the aroma characteristics of honey-like aroma (Sun et al., 2024). Keemun black tea has been attracted by consumer because of its high aroma and special taste (Guo et al., 2018). Lapsang Souchong is known for its smoky pine aroma (Li, Li, et al., 2024). Dianhong black tea is famous for its plump and firm appearance, outstanding golden trichome and rich flavor (Chen et al., 2022). Huangpu black tea presents a high sweet aroma and umami taste (Ouyang et al., 2024). Despite the distinct flavor profiles exhibited by various black tea products, limited systematic research has been conducted to scientifically characterize flavor compounds in different varieties of black tea commercially available in the same price range. This limitation hinders the improvement of quality and flavor control of black tea products, as well as the establishment of a scientific basis for consumers' purchasing decisions.

The quality of tea is largely determined by its taste and aroma (Ouyang et al., 2024). The taste of black tea is closely associated with the specific non-volatile metabolites (NVMs) of tea, mainly polyphenols, theaflavins and amino acids (Guo et al., 2018). It has been reported that the total catechin content in black tea leaves ranges from 27.98 mg/g to 74.00 mg/g (Aloo et al., 2024). Theaflavins constitute 3–5 % of the total extract of black tea and are generally regarded as a quality indicator for black tea (Muthumani & Kumar, 2007). The volatile metabolites (VMs) that influence the aroma characteristics of black tea include alcohols, aldehydes, esters, acids, hydrocarbons, and heterooxides (Su et al., 2022). The aroma characteristics of black tea are determined by the concentration, proportion, and category of these volatile compounds. Today, there are several chromatographic methods with various detectors in order to separate and detect NVMs and VMs. For example, gas chromatography (GC) with electron capture detector (ECD) and tandem mass spectrometry (MS/MS), ultra-performance liquid chromatography (UPLC) with photodiode array detector and ultraviolet detector (UV) have been used to quantify these chemicals (Bodur et al., 2025). Targeted quantitation techniques, which are crucial for the accurate quantification of target compounds in samples, have been extensively utilized in metabolomics and the quantification of substances (Chen et al., 2025). Headspace solid-phase microextraction (HS-SPME) is recognized for its exceptional ability to capture high boiling point volatiles, offering advantages in sensitivity, cost-effectiveness, and automation (Zhou, Qin, et al., 2025). Combined with the exceptional qualitative and quantitative potential of GC–MS, which makes HS-SPME-GC–MS particularly well-suited for the identification of VMs in tea. The technology of gas chromatography-olfactometry (GC-O) analysis was a forceful technique to exploit the aroma-active compounds from the complex mixture together with human sense (Li, Wang, et al., 2024). Zheng et al. (Zheng et al., 2023) studied the key aroma components and their relationship with sensory attributes in different aroma types of Fu brick tea according to HS-SPME combined with GC–MS, odor active value (OAV) and partial least-squares analysis (PLS) methods, they also found that GC-O combined with OAV method could minimize the errors of which individually performed by OAV method.

This study selected Keemun black tea (QM), Lapsang Souchong (ZS), Fengqing Dianhong (FQ), Zunyi black tea (ZY) and Huangpu black tea (HP) with a price range of 45–55 USD per kilogram as experimental materials. The UPLC, comprehensive two-dimensional gas chromatography quadrupole time-of-flight mass spectrometry (GC × GC-QTOFMS) and sensory evaluation methods were combined to conduct a comprehensive and systematic analysis of the quality of five different varieties of mid-range black teas. This study aims to provide a basis for research on the flavor characteristics and quality control of black tea, while also offering a scientific reference for consumers purchasing mid-tier commercial-grade black tea products.

## 2 Materials and methods

### 2.1 Source of tea samples

A total of 18 tea samples were collected, including four samples of Zunyi black tea, four samples of Keemun black tea, three samples of Lapsang Souchong black tea, three samples of Fengqing Dianhong black tea and four samples of Huangpu black tea. All tea samples, graded one bud and two leaves and priced at USD 45–55, were sourced from different companies. For each tea type, samples were produced from the same raw material variety. The specific sample information was shown in Table S1.

### 2.2 Flavor evaluation

#### 2.2.1 Traditional sensory evaluation

The sensory assessment was conducted by a panel consisting of seven well-trained assessors, which included three males and four females, with ages ranging from 25 to 30 years. National regulations do not mandate ethics approval or the establishment of a human ethics committee for sensory reviews. Before the commencement of the trials, all participants were informed of the specifics of the trial and voluntarily consented to participate. The traditional sensory evaluation was performed by the Chinese standard method (GB/T 23776–2018). Briefly, 3 g of black tea was steeped in 150 mL of boiling water for 5 min. Subsequently, the tea leaves were strained, and the panelists evaluated the quality and assigned scores accordingly.

#### 2.2.2 Quantitative descriptive analysis (QDA) of the aroma and taste

The QDA experiments conducted on the aroma profiles of black teas were performed by the same panel members who participated in the traditional sensory evaluation. Numerous standards were used to train the assessors to distinguish the corresponding flavor types and intensities. Caffeine (bitterness), epigallocatechin gallate (astringency), citric acid (sourness), theanine (umami), glutamic acid (mellow), and sucrose (sweetness) were used as standards with corresponding taste descriptors. Geraniol (floral), citral (fruity), ethyl vanillin (sweet), cedrol (woody), maltol (potato-like), and (Z)-3-hexene-1-ol (fresh) were used as standards with corresponding aroma descriptors (Zhou, Tian, et al., 2025). Ten concentrations were selected from a 1 to 1000 times the threshold concentration gradient of standards for the intensity training (within the safe range for human body). All assessors completed 60 h of training to identify, articulate, and differentiate various flavor characteristics. Prior to the final experiment, assessors must pass a completely blind test. They were required to accurately determine the type and intensity of flavor and complete the answer. Subsequently subjected the intensity values (0−10), taste descriptors (sweetness, umami, bitterness, astringency, sourness, annd mellowness), and aroma descriptors (fruity, floral, sweet, woody, green, and potato-like) of each sample infusion to a sensory test. The seven panelists utilized a scale from 0 to 10, as previously described in a study, to symbolize the intensity values, where 0 represented none or no perception and 10 indicated an extremely strong intensity (Zhang et al., 2024). The sensory attribute intensity was based on the average ratings given by the seven panelists.

### 2.3 The determination of macro-compounds, tea catechins, theaflavin components, Gallic acid and L-theanine

The aqueous extracts, flavones and total polyphenol of black tea were determined according to previous published method (Zhang et al., 2023). Free amino acid (FAA) was measured according to national standards (GB/T 8314–2013). The catechins, gallic acid(GA), and alkaloids were detected using high performance liquid chromatography (Shimadzu, Japan) according to previously methods (Lu et al., 2024). The theaflavin fractions were determined based on the national standards (GB/T 30483–2013).

### 2.4 The GC × GC-QTOFMS detection of volatiles

#### 2.4.1 Headspace solid phase micro-extraction for volatiles extraction

Accurately weighting of 0.5 g of black tea was done into 20 mL headspace vial. Then, 10 μL of ethyl decanoate (8.63 mg/L, 99.9 %, Anpel-Trace, Shanghai, China) internal standard solution was added. The headspace vial was equilibrated at 80 °C for 20 min, and an extraction head (50/30 μm SFIB-DVB/C-WR-80/10-P1, Agilent Technologies Inc., State of California, America) was inserted to adsorb the sample for 20 min, and then the sample was resolved at 250 °C for 3 min.

#### 2.4.2 Chromatographic conditions for GC × GC analysis

Modulation Column Type: SV(C6 ∼ C40, Agilent Technologies Inc., State of California, America). The one-dimensional (1D) and two-dimensional (2D) columns used were an HP-5 ms Ultra Inert (30 m × 0.25 mm × 0.25 μm, Agilent Technologies Inc., State of California, America) and a HP-5 ms-15 m + SV + 2D (2.32 m × 0.18 mm × 0.18 μm, Agilent Technologies Inc., State of California, America), respectively. The heating program was as follows: initially held at 40 °C for 1 min, The temperature was increased to 180 °C at 2 °C/min, and increased to 250 °C at 5 °C/min for 5 min. The carrier gas was helium (99.999 %) at a flow rate of 0.9 mL/min. The split ratio was 50:1, the inlet temperature was 250 °C, and the MSD transfer line temperature was 250 °C.

#### 2.4.3 Analysis conditions for tandem mass spectrometry

The acquisition process utilized full scan mode, a filament current of 5 μA. The ionization process employed an electron bombardment ion source (EI), with the electron bombardment source set at 70 eV and ion source temperature at 200 °C, and the temperature of the quadrupole was 150 °C.

#### 2.4.4 Qualitative and quantitative analysis of volatile compounds

The data obtained from GC × GC-QTOFMS detection were analyzed using Canvas Panel software, set the minimum peak detection signal-to-noise ratio to 10. NIST 20 standard spectral library and retention index (RI) were used for qualitative analysis. Samples were analyzed under the same chromatographic conditions as n-alkanes (C7 ∼ C25, purity 99 %, Shanghai Macklin Biochemical Co., Ltd., Shanghai, China) with forward and reverse injections, and retention index values were calculated for each compound in the samples, retaining compounds with forward and reverse matches greater than 700 and with RI deviation less than 15. The relative content (μg/g) of each VM was calculated using the internal standard method, taking into account the concentration (10 mg/L), addition amount, peak area of the internal standard, and the weight of the tea sample (Wen et al., 2023).

#### 2.4.5 Calculation of relative odor activity value (ROAV) of volatiles

To assess the contribution of VMs to the aroma profile of the tea samples, the ROAV of VMs was calculated. The ROAV was calculated by dividing the relative concentration of VMs (μg/g) by their odor threshold (OT) (μg/g) in water, which was obtained from references (Zhang et al., 2023) and VCF (https://www.vcf-online.nl/Vcf-Home.cfm).

#### 2.4.6 Analysis of odor types and intensities of volatiles using GC-O

The GC–MS system was connected to the olfactory evaluation port (ODP-4) for aroma analysis, and the detected components were fed into the detector and the sniffer in a 1:1 proportion. The temperature of the transmission line was 250 °C, and the temperature of the sniffing port was 150 °C. The parameters of the column warming procedure were the same as those of the abovementioned GC × GC conditions. The olfactory analysis team comprises three individuals selected from the QDA assessment panel, and was evaluated using a 1–4 grading system (Wen et al., 2023). Aroma Intensity (AI) was recorded using a removable resistor, where “1” indicates a weak odor, “2” a moderate odor, “3” means strong odor, “4” means strong odor, and the final assessment will be determined by at least “2” panelists sniffing similar odor descriptions and intensities at the same time stage, and the results of the three sniffs will be averaged.

### 2.5 Statistical analysis

Samples were subjected to principal component analysis (PCA), partial least squares discriminant analysis (PLS-DA), Venn plot, and clustering analysis using the Metware Cloud (MC), a free online platform for data analysis (https://cloud.metware.cn). One-way analysis of variance (ANOVA) was performed using SPSS 19.0. The radar chart, stacked graphs, and flavor wheel were plotted using Origin 2024 (OriginLab Corp., Northampton, MA, USA). The column chart and clustering heatmap were generated using GraphPad Prism software (version 8.3.0, San Diego, CA, USA).

## 3 Results and discussion

### 3.1 Sensory evaluation

To reduce the workload of follow-up testing and screen out representative black tea samples, we conducted sensory evaluations of four samples of Zunyi black tea, four samples of Keemun black tea, three samples of Lapsang Souchong black tea, three samples of Fengqing Dianhong black tea and four samples of Huangpu black tea respectively (Tables S1–2 through S1–7, Fig. S1). Representative samples of the highest sensory scores were used for follow-up studies and were named Zunyi black tea (ZY), Keemun black tea (QM), Lapsang Souchong black tea (ZS), Fengqing Dianhong black tea (FQ) and Huangpu black tea (HP).

**Fig. 1:**
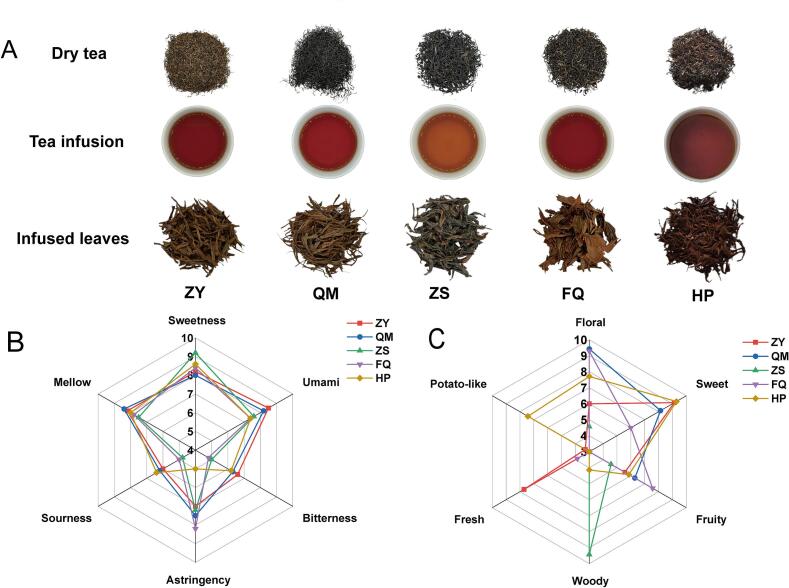
The sensory evaluation of the five different varieties of black teas. (A) The sensory evaluation forms and pictures; (B) and (C) Quantitative descriptive analysis of taste and aroma, respectively.

To macroscopically explore the flavor differences of the five different varieties of black teas, traditional sensory evaluation was performed on the representative samples, with the results presented in Fig. 1A. ZY was characterized by a wiry shape and a black bloom appearance, exhibiting a sweet and slightly fresh scent and a mellow and thick taste. QM featured a tight and heavy shape and a black bloom appearance, with a high and lasting scent marked by a floral aroma, as well as a sweet and mellow taste. ZS was coarse and robust in shape, with a brownish-auburn appearance, a unique smoky pine aroma and a mellow taste. FQ had a tight and heavy shape and a brownish-auburn appearance, with a lasting, pure, and normal aroma, as well as a slightly astringent, heavy, and thick taste. HP was tight and heavy in shape, black in appearance, sweet in aroma, heavy and thick in taste. The sensory scores of the five black teas ranged from 87 to 91. Although the sensory score increased with the price, the sensory scores and price ranges of the black teas in this study are insufficient to support the conclusion that tea pricing depends on quality. Further validation is required.

QDA is a widely used evaluation method for flavor analysis (Ouyang et al., 2024). The taste attributes of the five different varieties of black teas were mainly sweet, mellow and umami (Fig. 1B), with ZS having the strongest sweetness, ZY having the highest umami, and QM having the strongest mellow. Overall, QM has the highest taste score. Significant variations in aroma profiles were observed among the black tea samples through QDA. Specifically, ZY demonstrated predominant sweet notes, while QM exhibited the most intense floral characteristics. ZS displayed a distinctive pine-smoke aroma, distinguishing it from other samples. FQ presented a balanced aromatic profile combining floral and fruity aroma. Notably, HP showed a complex bouquet dominated by sweet aroma accompanied by complementary floral and potato-like aroma. There were great differences in the scores of aroma attributes among different black tea samples.

### 3.2 Overview of NVMs

**Fig. 2:**
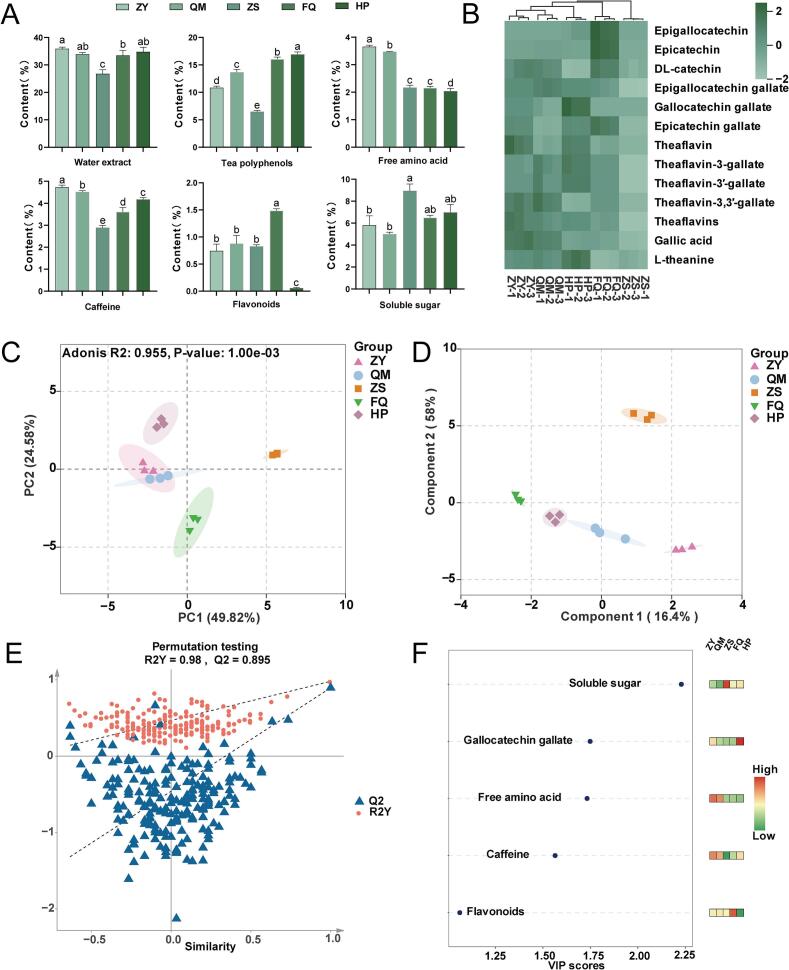
Differences of NVMs in the five different varieties of black teas. (A) The contents of water extract, tea polyphenols, free amino acid, caffeine, flavonoids, soluble sugar. Different letters indicate significant difference at the *P* < 0.05 level. (B) Quantitative results for 11 taste monomers. (C) PCA score scatter plot of the non-volatile metabolites (NVMs). (D) PLS-DA score scatter plot of the NVMs. (E) Permutation test plots of the NVMs. (F) Heat map of the values and contents of taste monomers with VIP value of >1.

Tea quality is governed by multifactorial determinants, with taste attributes serving as critical evaluation parameters, determined by the composition and concentration of NVMs. In order to elucidate the contribution of NVMs to the taste of five different varieties of black teas, 19 NVMs were quantitatively detected and comprehensively analyzed (Fig. 2A and B), encompassing macro-compounds, catechins, theaflavins, gallic acid and L-theanine (Table S2).

#### 3.2.1 Macro-compounds

The water extract content serves as a crucial indicator of the richness of tea infusion and is strongly correlated with the intensity of the tea taste (Huang et al., 2024). The water extract content in ZY, QM, FQ, and HP differed slightly but was significantly higher than that in ZS (Fig. 2A). This may be a significant reason why ZS has the lowest sensory evaluation and mellow attribute scores (Table S1). Polyphenols are the main secondary metabolites of tea and contribute significantly to the flavor and health benefits of tea (Ouyang et al., 2024). Polyphenols are the main source of bitterness in tea. ZS has the lowest bitterness score, while HP, FQ and QM have higher scores, which are related to their polyphenol content (Fig. 2A). The abundance of free amino acids significantly affects the umami and sweetness of tea and contributes to the formation of aroma quality (Zhang et al., 2019). The amino acid content of ZY and QM was significantly higher than that of other black teas. This contributes to their fresher taste. (Fig. 2A). Flavones play a significant role in enhancing the liquor color and intensifying the bitter and astringency taste in black tea. This results in the strongest FQ astringency attribute and the weakest HP astringency attribute (Fig. 1B). Of particular note is the extremely low level of flavonoids in HP (Fig. 2A), we speculated that this may be due to the unique characteristics of the tea plant variety. Additionally, caffeine is recognized as a key flavor compound in tea and contributes to bitterness (Hua et al., 2021). Soluble sugars are known to be the main components responsible for the sweet and thick tastes, in addition to being the basis for the sweet aroma (Alasalvar et al., 2012). The difference in soluble sugar content lead that ZS had the strongest sweetness attribute, while QM had the weakest (Fig. 1B and 2A). The lowest caffeine content and highest soluble sugar content contribute to the sweet and mellow taste of ZS.

#### 3.2.2 Tea polyphenols and catechins components

Catechins constitute the most abundant flavonoids in tea plants. Upon oxidation and polymerization, catechins are transformed into TFs, TRs, TB, and other high-molecular-weight polymers. These compounds significantly influence the sensory attributes of tea, including its taste and color (Fan et al., 2016). The simple catechins identified in this study encompass epigallocatechin (EGC), epicatechin (EC) and DL-catechin (DL-C), while the ester-type catechins comprise epigallocatechin gallate (EGCG), gallocatechin gallate (GCG), and epicatechin gallate (ECG). EC and EGC, EC and EGCG, EGC and EGC, EGC and EGCG were conjugated to form different theaflavins monomers, respectively (Hu et al., 2024). This may explain why EGC was not detected in ZY, QM and ZS, and GCG was not detected in QM, ZS and FQ (Table S2). FQ has a higher content of EGC, DL-C, EC, and ECG (Fig. 2B), which contributes to its slightly astringent, heavy and thick taste profile. The low content of EGC, EC, EGCG, GCG and ECG in ZS contributes to its sweet, mellow taste.

#### 3.2.3 Theaflavins

As one of the main active pigment and polyphenol in black tea, theaflavins can improve human health in many ways (Zhao et al., 2025). The main TFs, including theaflavin (TF), theaflavin-3-gallate (TF3G), theaflavin-3′-gallate (TF3′G), and theaflavin-3,3′ -gallate (TFDG). Except for the fact that theaflavins were not detected in ZS, there was no significant difference in theaflavins monomer content in other black teas (Fig. 2B). Theaflavins are intermediate products formed by the oxidation of tea polyphenols during the initial stage of black tea fermentation (Zhao et al., 2025). If fermentation is excessive, theaflavins undergo further oxidation to produce thearubigins and theabrownins. In the traditional process of Lapsang Souchong, the “pan-firing” (high-temperature stir-frying) and “smoking and drying” (pine smoke) steps may accelerate oxidation, resulting in a large proportion of theaflavins being converted into thearubigins and theabrownins, which significantly reduces their content. Meanwhile, the smoking step may reduce the stability of theaflavins. The chemical components in pine smoke may bind to theaflavins, or high temperatures may promote their degradation. The high content of theaflavins has an important influence on the color and quality of black tea (Zhao et al., 2025).

#### 3.2.4 Gallic acid and L-theanine

Gallic acid (GA) and caffeine are important contributors to the sourness and bitterness of black tea (Wang, Chen, et al., 2022). The GA content of different black teas varied significantly, with ZY having the highest GA and caffeine content, which may be an important source of astringency properties of ZY (Fig. 2A and B). Whereas, ZS has the lowest GA and caffeine content (Fig. 2A and B). This is a key reason why ZS has low sourness and bitterness scores. L-Theanine is a non-protein amino acid that contributes to the umami property of tea and can regulate protein metabolism of healthy organisms (Lin et al., 2022). The content of theanine in ZS and FQ was lower, and the content of theanine in HP was higher (Fig. 2B), which was consistent with the results of umami properties scores.

#### 3.2.5 Multivariate statistical analysis of the differentia NVMs

The principal component analysis (PCA) map effectively illustrates the abundance of NVMs in the samples. The closer the samples are on the map, the more similar they are (Ma et al., 2024). We observed that the five different varieties of black teas could be clustered separately, with a clear distinction among the groups (Fig. 2C). To further identify the key macro-components that differentiate the tastes between the 5 types of black tea, partial least squares discriminant analysis (PLS-DA) was employed on 17 NVMs (Fig. 2D). Cross-validation analysis showed that the PLS-DA models were reliable (Fig. 2E). We can know from Fig. 5F that the variable importance in projection (VIP) value of soluble sugar, gallocatechin gallate, free amino acids, caffeine, and flavonoids is greater than 1 (Fig. 2F), and metabolites with VIP > 1 are generally considered to be significantly different (Zhang et al., 2023).

### 3.3 Overview of VMs

#### 3.3.1 Identification and quantitative analysis of VMs

**Fig. 3:**
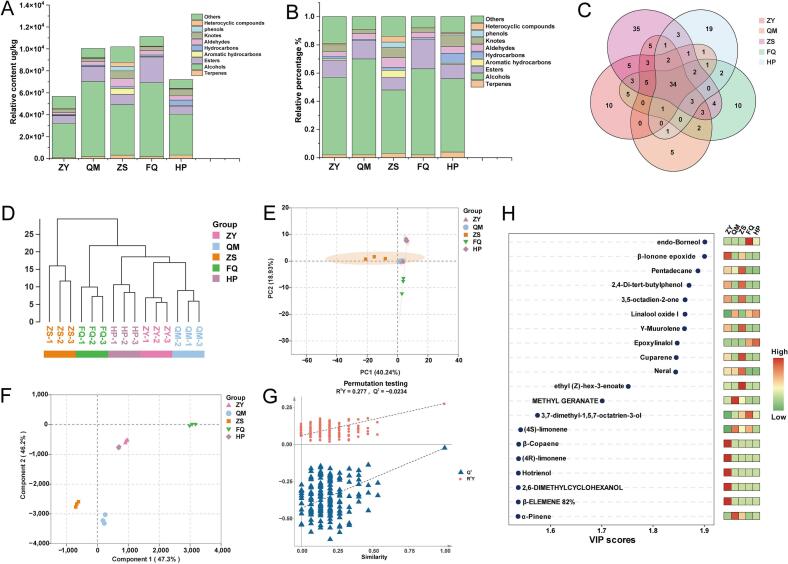
Differences of VMs in the five different varieties of black teas. (A) and (B) Relative content and percentage of different types of volatile metabolites (VMs) in tea samples; (C) Venn diagram of VMs; (D) The cluster analysis of five black tea samples; (E) PCA score scatter plot of the VMs; (F) PLS-DA score scatter plot of the VMs; (G) Permutation test plots of the VMs; (H) Heat map of the values and contents of VMs with the top 20 VIP value of >1.

A total of 165 VMs were identified across all samples (Table S3), including 32 terpenes, 28 alcohols, 27 esters, 16 aromatic hydrocarbons, 16 hydrocarbons, 16 aldehydes, 12 knotes, 8 phenols, 6 heterocyclic compounds, and 4 others. Most of these VMs are degradation products of carotenoids, fatty acid derivatives, phenylpropanoids, benzene ring compounds, and terpenoids present in the tea leaves (Wang, Li, et al., 2022). Alcohols had the highest average percentage of total aroma compounds across all groups, exceeding 50 % (Fig.3. A and B). Esters averaged about 11 %, the other 8 classes of compounds were relatively consistent among the groups, averaging about 38 % (Fig.3. A and B). Alcohols and esters play an important role in the aroma of tea, often reflecting floral and fruity aromas (Zhu et al., 2021). It is worth noting that the average percentage of caffeine in all samples to the total aroma was as high as 11 % (Table S3). Song et al. (Song et al., 2025) found that caffeine has significant synergistic effect with flowery/fruity compounds, which can enhance the flowery/fruity aroma of key compounds in water.

With ZS containing the highest number of aroma compounds (108), followed by FQ (74), ZY (72), HP (70) and QM (64). Except for ZS, there was no significant difference in the amount of VMs in the other samples (Fig. 3C). The unique smoking procedure leads to the richest VMs varieties and fascinating sensory characteristics of ZS (Li, Wang, et al., 2024). All samples shared 34 VMs (Fig. 3C). The shared VMs were dominated by terpenes and knotes, these substances may be an important basis for the formation of the characteristic aroma of black tea. The cluster plot showed that the samples were reproducible (Fig. 3D). From the PCA (Fig.3E), ZY and QM were found to overlap, indicating that they have similar VMs compositions, which was consistent with the clustering results. This may be due to similar processing varieties and processing techniques. The clear difference among the other samples suggested that the tea samples had different VMs profiles.

#### 3.3.2 Discrimination of VMs in the five different varieties of black teas

To further distinguish the five different varieties of black teas and identify the discriminatory VMs, PLS-DA analysis was performed (Fig. 3F). A cross-validation analysis showed that the PLS-DA model was reliable. The Q^2^ regression line intersects the vertical axis below zero, indicating that there is no overfitting, validating the effectiveness of the model (Fig. 3G). A VIP ≥ 1.0 and *p* < 0.05 were defined as the two conditions for discriminating VMs in the five different varieties of black teas. On the basis of these criteria, a total of 51 discriminatory VMs were identified (VIP ≥ 1.0, *p* < 0.05) (Table S4). The top 10 compounds were borneo, *β*-Ionone epoxide, pentadecane, 2,4-Di-tert-butylphenol, 3,5-octadien-2-one, linalool oxide I, *γ*-muurolene, epoxylinalol, cuparene, and neral. There were differences in the concentrations of the discriminatory VMs among the five different varieties of black teas (Fig. 3H). The variations in the types and concentrations of these compounds could potentially be used to assess the quality of five different varieties of black teas. Borneo has the highest VIP value (1.9011, Table S4) and has the scents of rosin and mint, but it is less studied in tea. However, borneol is the featured ingredient in *Camphora Fabr.* species, widely used in the pharmaceutical and cosmetic industries (Zhang et al., 2025). And the content of Borneo in FQ is much higher than that of other samples (Table S3), which may be a characteristic aroma-active compound in FQ that contributes to its woody and fruity aroma properties. Among the discriminatory VMs, linalool oxide I (sweet, creamy, floral) had the highest average concentration in each of the five different varieties of black teas (34.06 μg/kg, 197.17 μg/kg, 64.14 μg/kg, 180.12 μg/kg, and 217.29 μg/kg, Table S3). Linalool oxide I is a common volatile component in tea and always contributes positively to the formation of the sweet and floral aroma of black teas (Cui et al., 2024). In tea, linalool oxide I exists in free and glycoside bound forms and is synthesized and accumulated mainly during the withering stage of tea manufacturing (Xiao et al., 2022). Borneo (rosin, mint; detected in FQ and HP), *β*-ionone epoxide (fruity, woody, floral; unique to ZY and ZS), 3,5-octadien-2-one (fruity, green; exclusive to ZY), and neral (sweet, lemon; present in ZY, QM, and ZS), were not universally detected across all samples (Table S3). These absence/presence patterns contributed significantly to the formation of distinctive aroma profiles among black tea varieties.

#### 3.3.3 Aroma-active compounds in the five different varieties of black teas

The aroma profile of tea depends on the proportion, content, and ROAVs of VMs. The ROAV>1 is a common indicator to evaluate the important contribution of substances to the aroma of tea. The statistics of ROAV >1 in the volatile profiles were provided in Table S5. A total of 41 VMs with ROAV greater than 1 were identified, among which seven exhibited ROAV consistently exceeding 1 across all analyzed samples. These 7 VMs were (*E*)-furan linalool oxide, linalool, benzeneacetaldehyde, β-cyclocitral, geraniol, methyl salicylate, and *α*-Ionone (Table S5). (*E*)-furan linalool oxide with floral aroma and methyl salicylate with mint aroma were identified as essential aroma-active compounds in the formation of basic Congou black tea aroma (Mao et al., 2018). Linalool is a representative aroma compound commonly found in tea (Xiao et al., 2022) that makes a unique contribution to the floral aroma of black tea (Lu et al., 2024). Benzeneacetaldehyde is noted for its floral and honey-like aroma in tea, which is converted from phenylethanol during the fermentation of black tea (Aaqil et al., 2024). β-cyclocitral, which also plays an important role in tea aromas, presents a fruity aroma and was produced due to the primary oxidation of β-carotene during the fermentation or drying stages (Yang et al., 2024). Geraniol provides rose and geranium aromas for Zunyi black tea, which greatly contributes to the characteristic aroma of black tea (Sun et al., 2024). And the *α*-ionone contributed violet and woody odor (Wang, Shen, et al., 2022). The formation of the basic aroma characteristics of the five black teas was founded on these VMs with a common ROAV value greater than 1.

The 17, 20, 36, 19 and 14 VMs with ROAVs greater than 1 were detected in ZY, QM, ZS, FQ and HP, respectively. These differences in number contributed to the variation in aroma characteristics between the five different varieties of black teas. The large number of ROAV >1 VMs that ZS has may be an important reason for its rich, balanced aroma formation. P-cresol (smoky, herbal) and 4-ethyl-2-methoxyphenol (smoky) that are unique to ZS and three woody compounds ((*E*)-linalool oxide (pyranoid), guaiacol, *trans*-calamenene) contribute to the unique smoky aroma of ZS (Table S5). Of the VMs in ZY, five have ROAV values greater than 100: linalool (orange and floral, ROAV = 917.28)， geraniol (floral and fruity, ROAV = 615.00)， *trans*-calamenene (woody, ROAV = 369.62)， *β*-ionone epoxide (fruity, woody and floral, ROAV = 156.72)， and cedrol (sweet and woody, ROAV = 104.23) (Table S5). These VMs mainly show floral and fruity aromas. However, the QDA results suggest that ZY is dominated by a sweet aroma. This may imply that the tea's aroma is not expressed as a single substance, and that there are potential masking or synergistic effects between different substances. Of the VMs in the QM, three had ROAV values greater than 100: geraniol (floral and fruity, ROAV = 3764.88), *trans*-calamenene (woody, ROAV = 798.56), and and linalool (orange and floral, ROAV = 670.31) (Table S5). It is worth noting that the ROAV value of geraniol in QM is much higher than in other samples, which contributes to its strong floral attributes. Substances with ROAV values greater than 100 in FQ include linalool (orange and floral, ROAV = 2001.83) and geraniol (floral and fruity, ROAV = 1156.03). In HP, substances with ROAV values greater than 100 include *trans*-calamenene (woody, ROAV = 1033.32), linalool (orange and floral, ROAV = 835.06) and geraniol (floral and fruity, ROAV = 755.399). The key substances with high ROAV values are similar across different varieties of black tea, so differences in aroma were mostly determined by differences in content and the synergy between substances.

By considering the PLS-DA results and ROAVs together, a total of 10 compounds were identified as aroma-active compounds (VIP > 1.0, *p* < 0.05 and ROAV >1) in the five different varieties of black teas (Table S6). Among the top five substances with the highest ROAV values, four were identified as linalool and its derivatives, specifically linalool oxide I, (*E*)-furan linalool oxide, linalool, and (*E*)-linalool oxide (pyranoid). Linalool and its derivatives were mainly synthesized through the terpenoid synthesis pathwaym (Zhou, Tian, et al., 2025). (*E*)-furan linalool oxide (floral) and (*E*)-linalool oxide (pyranoid) (floral, woody) were identified as characteristic aroma components of Congou black tea and ripe Pu-erh tea, respectively (Mao et al., 2018; Tian et al., 2024). Linalool and phenylethyl alcohol have high concentrations in QM and are the key aroma components of Keemun black tea (Table S6), which is consistent with previous study (Xu et al., 2023). Heptane and *β*-ionone epoxides were only detected in FQ and were the markers of FQ (Table S3), giving it a floral and fruity aroma. Heptanal, which derived from lipid degradation, has a fatty, herbal, and nutty odor (Lu et al., 2024; Zhou et al., 2025). *β*-ionone epoxide, a carotenoid monoterpene with epoxy groups at positions 5 and 6, has a sweet berry and woody aroma, formed from oxidation of *β*-ionone (Hao et al., 2025). The *α*-cubebene with herbal odor was only detected in QM only and can be used as a marker of QM (Table S6). The low threshold (0.00002 μg/kg) of *trans*-calamenene allows it to make an important contribution to the aroma (Table S6).

#### 3.3.4 GC-O analysis of the five different varieties of black teas

Using the ROAV value as an index to select key aromatic substances has certain limitations because it has not been verified by direct experimentation. To study the aroma-active compounds of five different varieties of black tea in more detail and validate the ROAV results from other perspectives, the samples were analyzed using GC-O technology. A total of 51 aroma-active compounds were detected and identified, where the five different varieties of black teas, ZY, QM, ZS, FQ, and HP, had 21, 22, 37, 25, and 22 aroma-active compounds, respectively (Table S7). Geraniol (floral, fruity; intensity = 3.33, 3.00, 3.33), linalool (floral, sweet; intensity = 3.00, 2.67, 3.33), and phenylethyl alcohol (rose, intensity = 3.00, 3.00, 3.00) in ZY, QM, and FQ had high aroma intensities. These substances with high olfactory intensity contribute to the formation of the floral properties of ZY, QM and FQ. Linalool (sweet, floral), linalool oxide I (sweet, creamy, floral), (*E*)-furan linalool oxide (sweet, creamy, floral), and *α*-ionone (sweet, floral), constituted the top six olfactory intensities aroma-active compounds in sample HP. This compositional dominance mechanistically explains HP's distinctive superiority in floral aroma intensity among the investigated black tea samples. The highest olfactory intensities in ZS were *β*-ionone (floral; intensity = 2.67), geraniol (floral, fruity; intensity = 2.67), and phenylethyl alcohol (rose, intensity = 2.67). Comparative analysis revealed that ZS and HP exhibited lower overall olfactory intensities compared to other samples. Notably, ZS demonstrated balanced aroma characteristics despite its reduced odor potency, as evidenced by its diversified profile containing 8 floral, 7 fruity, 9 sweet, and 5 woody aroma-active compounds. 4-Ethyl-2-methoxyphenol is an important aroma-active compounds of the smoky odor of ZS, which was derived from ZS's special smoking process (Yao et al., 2005). Meanwhile, profound, rich, and intense woody aroma is also an important feature of ZS (Su et al., 2025). Moreover, 30 out of 41 species with an ROAV value greater than 1 were smelled. Many volatile compounds, such as geraniol, linalool, and *α*-Ionone showed high values both in GC-O analysis and ROAV, which suggested a high positive relationship between these two methods. However, there were some differences in these results which obtained by the two methods. The assessors did not detect the eleven substances with an OAV greater than one, possibly because the GC-O analysis was influenced by their subjective judgment. Additionally, some substances with ROAV values below 1 exhibit an obvious odor intensity in GC-O analysis. For example, *β*-ionone, *α*-terpineol, and ethyl hexadecanoate, this difference was partly due to these compounds might have very high odor thresholds or odor-active compounds binding matrices (Zheng et al., 2023). These findings suggest that using ROAV and GC-O together can improve the accuracy of screening results. Noteworthily, the methods consider each compound individually, but ignored the interactions in aroma compounds. To explore the interaction mechanism between these key aroma compounds, we will conduct aroma recombination and omission tests in the future.

**Fig. 4:**
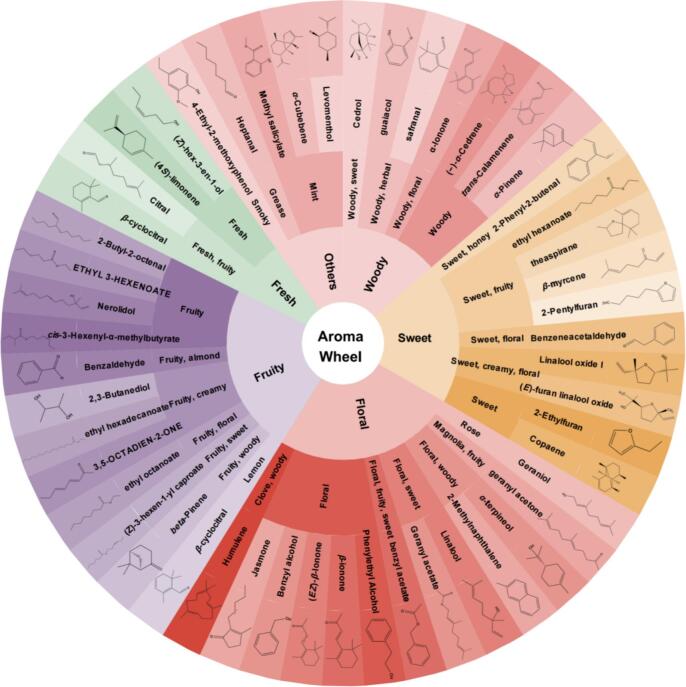
Aroma wheel for the five different varieties of black teas aroma types consisting of four layers. The first through fourth layers are the classification, aroma descriptors, names, and structural formulas of the aroma compounds, respectively.

Constructing a standardized flavor wheel for sensory evaluation of tea is an important strategy for product quality control (Liang et al., 2024). The 51 aroma-active compounds were divided into six categories according to the aroma description, woody: 7, sweet: 10, fruity: 12, fresh: 4, floral: 13, and other: 5. These results were used to construct the aroma wheel of five different varieties of black teas (Fig. 4) to visualize the composition and structure of aroma-active compounds in five different varieties of black teas. The synergistic interaction of these odor-active compounds constitutes the foundational framework for the distinctive aromatic profiles observed among black tea varieties. The aroma wheel developed in this study, which systematically categorizes aroma-active compounds based on their molecular structural features and corresponding olfactory characteristics, serves as a foundational guide for establishing a quantitative evaluation standard for the aroma quality of black tea.

#### 3.3.5 Key aroma-active compounds in the five different varieties of black teas

**Fig. 5:**
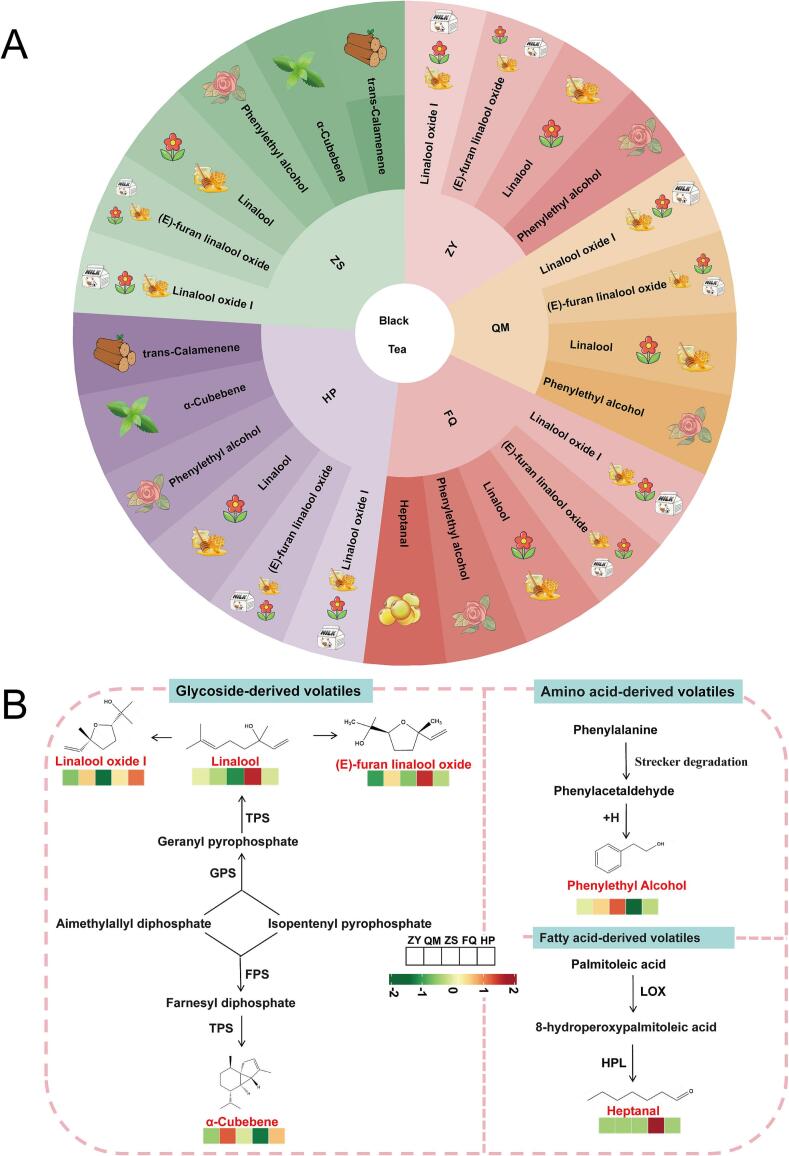
The 7 key aroma-active compounds in the five different varieties of black teas. (A) Aroma wheel of key aroma-active compounds for the aroma characterization of the five different varieties of black teas aroma types; (B) Biosynthetic pathway of key aroma-active compounds in five different varieties of black teas. GPS: geranyl pyrophosphate synthase; FPS: farnesyl diphosphate synthase; TPS: terpene synthase; LOX: lipoxygenase; HPL: hydroperoxide lyases.

Compounds with VIP > 1 are important for distinguishing between different samples. ROAV >1 screens for compounds with significant aromatic effects from the olfactory threshold level. GC-O analysis can detect aromatic compounds that can be smelled directly. Prior investigations have demonstrated that GC-O combined with ROAV could minimize the errors of which individually performed by GC-O method (Zheng et al., 2023). Further combination with VIP > 1 can identify key aroma-active compounds that distinguish between different black tea samples. To clarify the key aroma-active compounds of different black tea samples, the VIP values, ROAV values and GC-O analysis results were combined. The 7 key aroma-active compounds were screened, comprising linalool, phenylethyl alcohol, linalool oxide I, *α*-cubebene, (*E*)-furan linalool oxide, *trans*-calamenene, and heptanal (Table S8). Consequently, 4, 4, 6, 5, and 6 aroma-active compounds were identified for ZY, QM, ZS, FQ, HP, and respectively. We used these key aroma-active compounds to construct an aroma-flavor wheel of five different varieties of black teas to visualize the contribution of these key aroma compounds to the aroma characteristics of the five different varieties of black teas (Fig. 5A). These seven key aroma compounds are mainly floral and sweet aromas, in addition, *α*-cubebene has a minty scent, *trans*-calamenene has a woody aroma and heptaneal has a fatty and herbal scent.

On the basis of literature search, the metabolic pathways of key aroma-active compounds were sorted. Fig. 5B summarizes the biosynthetic pathways of several key aromatic active compounds in black tea based on literature results (Ouyang et al., 2022; Wang et al., 2024; Yao et al., 2024). Glycoside-derived volatiles mostly existed in the form of bound states in fresh tea leaves. In the process of tea processing, hydrolysis reactions occur under the action of biological enzymes, light and temperature, resulting in the formation of free monoterpene alcohols (linalool) and aromatic alcohols (phenylethyl alcohol), which in turn affect the aroma quality of tea (Hua et al., 2024). As a typical representative of glycoside-derived volatiles, linalool is characterized by a floral and woody aroma (Li, Wang, et al., 2024). It is derived from geranyl pyrophosphate released by linalool synthase (Wang et al., 2024). In addition, linalool oxide I and (*E*)-furan linalool oxide were derived from the oxidative polymerization of linalool under continuous high temperature conditions (Zeng et al., 2021). The *α*-cubebene was considered aromatic VMs that contributed to the cinnamon odor of *C. cassia*, through its strong herbal and woody aromas. The synthesis pathway of it as follows: aimethylallyl diphosphate and isopentenyl pyrophosphate reacted enzymatically under the action of farnesyl diphosphate synthase to form farnesyl diphosphate, and then *α*-cubebene was derived from geranyl pyrophosphate released by terpene synthase (Yao et al., 2024). Amino acid-derived VMs are generally derived from the deamination, Strecker degradation, and Maillard reaction of amino acids (Ho et al., 2015). Phenylethyl alcohol was high in floral aroma of black tea and was considered to be important components for floral aroma (Yin et al., 2022). Linalool, linalool oxide, and phenylethyl alcohol are the main sources of black tea floral and fruity aromas (Lu et al., 2024), this is consistent with this study. Heptanal was considered the fundamental odorants associated with the flavors of citrus blend black teas (Wang et al., 2020). The main formation pathway of heptanal is as follows: palmitoleic acid is oxidized by lipoxygenase to produce 8-hydroperoxy palmitoleic acid, which is then converted by hydroperoxide lyase (Wang et al., 2024).

## 4 Conclusions

The characteristic flavor and chemical basis of the five different varieties of black teas with medium commercial quality were investigated. Sensory evaluation demonstrated significant differentiation among the samples: ZY demonstrated peak freshness with predominant sweet aroma, while QM characterized by mellow mouthfeel and concentrated floral bouquet. ZS displayed a distinctive smoky pine aroma, with FQ which presented pronounced astringency with floral-fruity aroma. HP showed sweet mellowness and multi-layered aroma. Soluble sugar, gallocatechin gallate, free amino acids, caffeine, and flavonoids were identified as characteristic taste components. Through GC-O analysis of 51 aroma-active compounds, a aroma wheel was constructed based on 6 aroma types of black tea. Integrating VIP, ROAV, and GC-O analytical approaches, seven pivotal aroma-active compounds were definitively characterized as the core aroma determinants across five varieties of black teas. Meanwhile, the synthesis pathways of key aroma-compounds were clarified. Future research should conduct systematic aroma recombination and omission experiments to elucidate the quantitative contributions in shaping the characteristic flavor profiles of different black teas.

The successful application of VIP, ROAV and GC-O analysis to the screening of aroma-active compounds has established a universal methodological framework applicable to diverse black tea varieties. The aroma wheel helps consumers to understand the aroma characteristics of different types of black tea more intuitively. The results provide a basis for the study of flavor property and quality control of black tea, and laid a foundation of investigating the characteristics of black teas from different cultivars.

## CRediT authorship contribution statement

**Mengcong Zhang:** Writing – review & editing, Writing – original draft, Visualization, Methodology, Investigation, Conceptualization. **Xiaoshuang Lu:** Writing – original draft, Validation, Investigation, Formal analysis, Data curation. **Peidi Zhang:** Methodology, Investigation. **Guangcai Li:** Methodology, Investigation. **Dongfang Li:** Investigation. **Han Xie:** Investigation. **Jian'’an Huang:** Investigation. **Sheng Zhang:** Investigation, Data curation. **Zhonghua Liu:** Investigation, Data curation. **Ailing Liu:** Writing – review & editing, Supervision, Funding acquisition, Conceptualization.

## Declaration of competing interest

The authors declare that they have no known competing financial interests or personal relationships that could have appeared to influence the work reported in this paper.

## Data Availability

Data will be made available on request.
