# Supplementary material for: Reveal the differences in flavor of five varieties of black teas via sensory evaluation, UPLC, and GC × GC-QTOFMS approaches
Source: Food Chem X. 2025 Oct 3;31:103083. doi: 10.1016/j.fochx.2025.103083 (PMC12538472; doi:10.1016/j.fochx.2025.103083)
Supplement: Supplementary material 1 [file mmc1.docx]

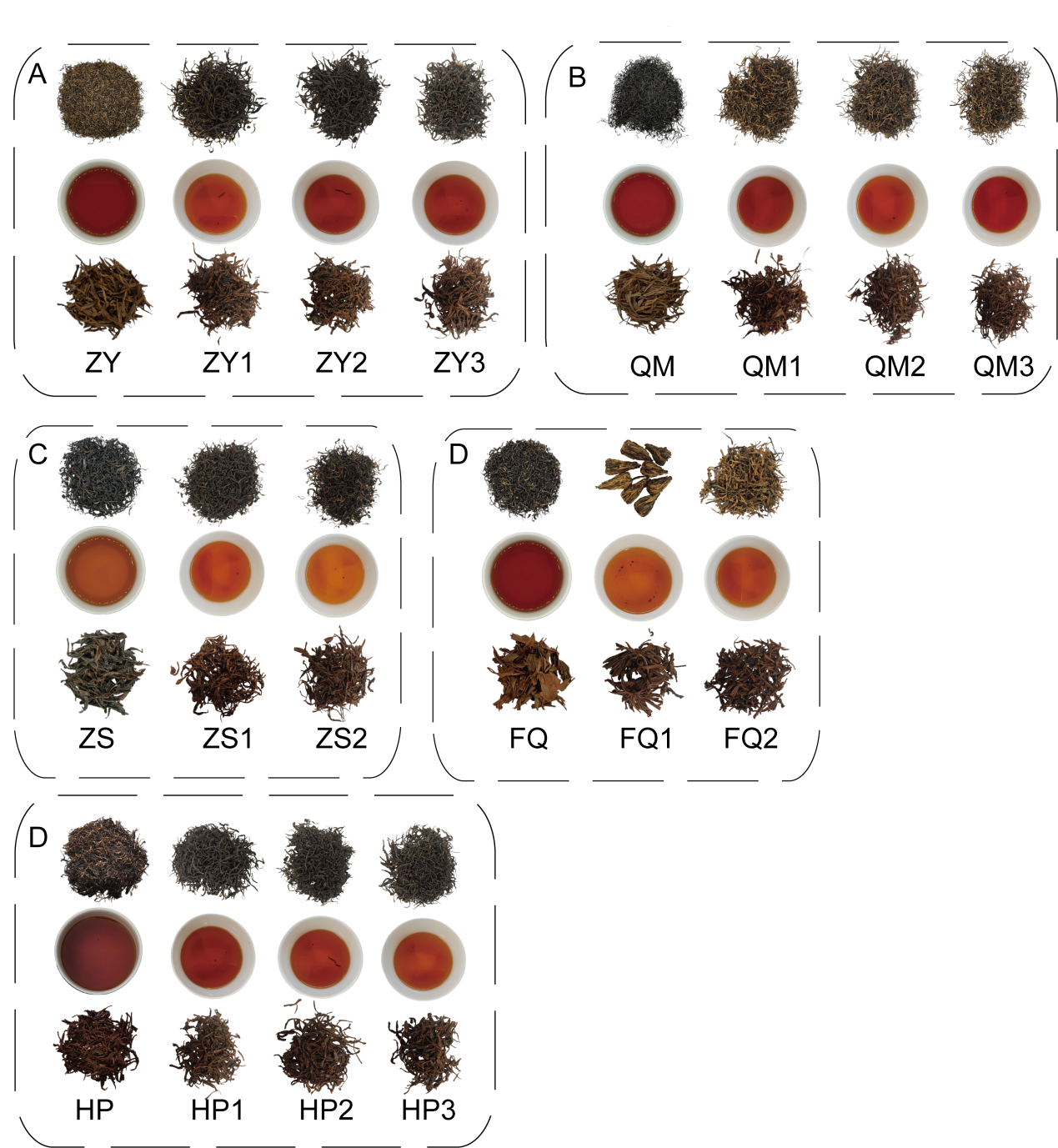


**Figure S1.** The sensory evaluation pictures of all tea samples. (A) Zunyi black teas. (B) Keemun black teas. (C) Lapsang Souchong black teas. (D) Fengqing Dianhong black teaS. (E) Huangpu black teas.


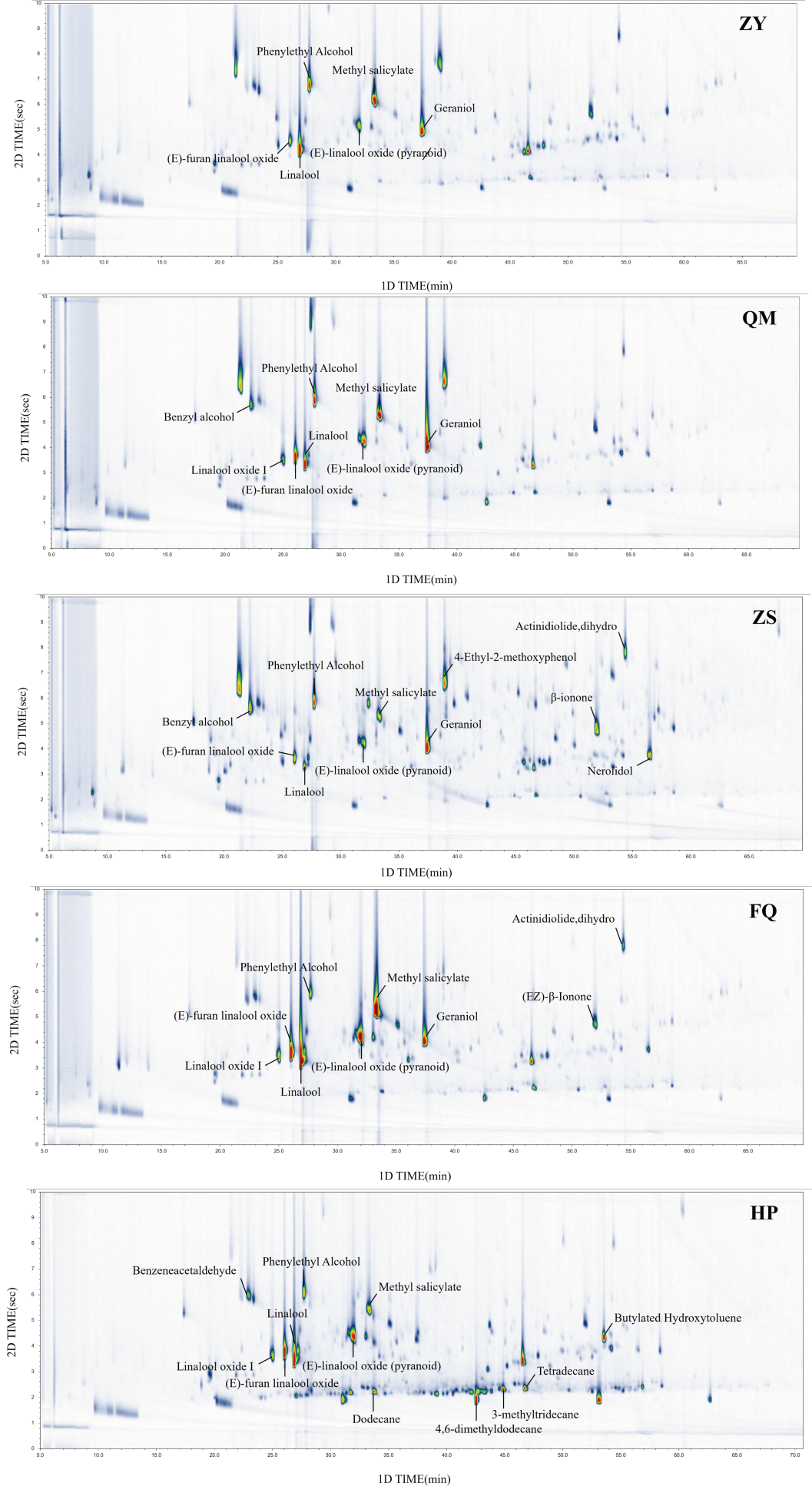


**Figure S2.** The GC-MS chromatograms for five varieties of black teas
